# Evolutionary trend of bovine β-defensin proteins toward functionality prediction: A domain-based bioinformatics study

**DOI:** 10.1016/j.heliyon.2023.e14158

**Published:** 2023-03-04

**Authors:** Saiful Islam, Mst Rubaiat Nazneen Akhand, Mahmudul Hasan

**Affiliations:** a Department of Physiology, Sylhet Agricultural University, Sylhet-3100, Bangladesh; b Department of Biochemistry and Chemistry, Sylhet Agricultural University, Sylhet-3100, Bangladesh; c Department of Pharmaceuticals and Industrial Biotechnology, Sylhet Agricultural University, Sylhet-3100, Bangladesh

**Keywords:** β-defensin, Bovine, Human, Cow, Disulfide, Domain

## Abstract

Defensins are small cationic cysteine-rich and amphipathic peptides that form of three-dimensional β-strand structure connected by disulfide bonds. Defensins form key elements of the innate immune system of multicellular organisms. They not only possess broad-spectrum antimicrobial activity but also have diverse roles, including cell signaling, ion channel agitation, toxic functions, and enzyme inhibitor activities in various animals. Although the role of β-defensins in immune responses against infectious agents and reproduction could be significant, inadequate genomic information is available to explain the whole β-defensin repertoire in cattle. No domain or motif-based functional analyses have been previously reported. In addition, how do defensins possess this magnitude of functions in the immune system is still not clear. Our present study, therefore, investigated the sequence divergence and evolutionary relations of bovine defensin proteins with those of humans. Our domain-based evolutionary analysis revealed four major clusters with significant domain variation while reserving a main antimicrobial activity. Our study revealed the β-defensin domain as the ancestor domain, and it is preserved in the first group of defensin protein with no α-helix in its structure. Due to natural selection, some domains have evolved independently within clusters II and III, while some proteins have lost their domain characteristics. Cluster IV contains the most recently evolved domains. Some proteins of all but cluster I might have adopted the functional characteristics of α-defensins which is largely absent in cattle. The proteins show different patterns of disulfide bridges and multiple signature patterns which might render them specialized functions in different tissue to combat against various pathogens.

## Highlights

• 64 β-defensins of bovine form four different clusters in domain-based phylogeny.
• Different patterns of disulfide bridges are observed in the bovine β-defensins.
• Though ruminants are largely devoid of α-defensins, presence of short α-helix structure in 3D model of some β-defensins in cluster II, III and IV indicates the adoption of some functional features of α-defensins.
• Domain variations and presence of different signature motifs are key to functional variation while conserving antimicrobial activites.

## Graphical abstract

**Figure:**
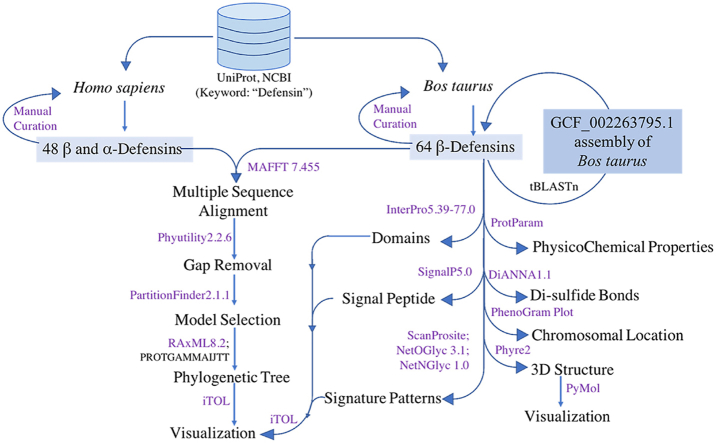

## Abbreviations

B_DEFB: Bovine β-Defensin
H_DEFB: Human β-Defensin
LAP: Lingual antimicrobial peptide
TAP: Tracheal antimicrobial peptide
EAP: Enteric β-Defensin
NMS: N-myristoylation site
MARCKS: myristoylated alanine rich C kinase substrate
PKC: protein kinase C
CK2PS: Casein kinase II phosphorylation site
NGS: N-glycosylation site

## 1 Introduction

Cow (*Bos Taurus*) is one of the key components of livestock that belongs to the Bovidae family. Cow are reared for good quality meat, milk, leathers, draft power, bone, manure, and a source of income to farmers [1]. Among the various constraints to cattle production, different types of bacterial, viral, parasitic, and fungal diseases are the most important which lessen the productivity of these animals [2]. The body defense mechanism is a vital factor that protects the body against any type of infection and this is associated with numerous agents, among which defensin proteins might play a crucial role behind this strong defense mechanism [3].

Defensins are small (29–35 amino acid), cysteine-rich cationic (1 to 5 positively charges), and amphipathic peptides that form three-dimensional β-strand structures connected by disulfide bonds [[4], [5], [6]]. Defensin molecules act as the key elements of the innate immune system of the body. Defensins are referred to as antimicrobial peptides (AMRs) or host defense peptides (HDPs), extensively dispersed in nature, produced by vertebrates to invertebrates, plants, and fungi [6]. Defensins are produced by various types of cells of the innate immune system, epithelium cells in animals, and different tissues in plants and fungi [7]. The main source of defensins produced is neutrophils and epithelial cells, but macrophages, dendritic cells, monocytes, and lymphocytes also produce these types of peptides [8]. There are various types of defensins produced by living organisms, which act as antimicrobial activities of phagocytic, the skin, and the mucosa [8].

Defensins not only act as broad-spectrum endogenous antibiotics activity that protects the animals against any types of infections associated with pathogenic microorganisms, ranging from bacteria to fungi and viruses, but also display a range of activities including inflammation, immunity, and wound repair [9,10]. They act as direct antimicrobial action, immune signaling, or both. They kill bacteria in many ways such as creating voltage-dependent channels in bacterial membranes that permit the inflow of water, and followed by elevated osmotic pressure ultimately splits the membrane of bacteria [11]. However, other defensins directly move through bacterial cell walls, bind to the target cells, and interrupt normal metabolic actions of bacteria. In viral infections, defensins might be interfered viruses by accumulating particles, deterring the binding of receptors, preventing virus penetration, uncoating particle, intracellular trading, the blockage of either cell signaling or gene expression. Defensins also attract immune cells and modify cell-mediated immunity. Therefore, defensins provide the barrier of first host defense fighting wide-ranging microbes, along with linkage of the innate and cell-mediated immune response [6].

Defensins have been classified into two super-families, *cis*-defensins and *trans*-defensins, and also contain multiple families [12]. The *cis*-defensins are found in invertebrates, fungi and plants, and the *trans*-defensins are found in humans and other vertebrates along with some invertebrates [[13], [14], [15]]. The super-families and families are determined by the overall tertiary structure of peptides; a strong pattern of disulfide bonds is detected in each family. The underlying genes of all families responsible for producing defensin are highly polymorphic.

According to the pattern and size of intramolecular disulfide bonds, vertebrates produced defensins are categorized into three subfamilies: α (alpha), β (beta), and θ (theta) [16,17]. All three types of defensins have six converted cysteines, such as: α-defensins contain C1–C6, C2–C4, and C3–C5 disulfide-linkages, but in β-defensins, they are connected by C1–C5, C2–C4, and C3–C6 disulfide bridges [18]. α-defensins are mainly produced by leukocytes and paneth cells of the gut in mammals [4]. θ-defensin, a cyclic peptide containing three pairs of disulfide bonds, is thought to ascend from peptide joining of couple hemi θ-defensin [4] and was initially detected from the white blood cells of rhesus monkey [19]. There is a highly conserved γ-core that is present in either α–γ, β–γ, γ–α, γ–β, α–γ–β or other conformation [57] in cysteine containing antimicrobial peptides including defensins. It has been found that this signature motif contains mainly GXC-X_3-9_-CC sequence in mammalian defensins [57,58]. This structural signature plays a significant role in host-pathogen defense mechanism.

β-defensins primarily express on epithelial cells of the skin, bronchial tree, the tongue, eyes, nose, and the urogenital tract of males and females [20,21]. The beta-defensin is encoded by two exons-genes. An inactive precursor (pre-propeptide), which is the main translated product, creates the signal of N-terminal sequence that is a short pro-piece, and a mature C-terminal peptide is cleaved from the pro-piece [22]. The initial exon translates the signal sequence, the second exon converts the propeptide and the mature peptide [22]. β-defensins were detected in various vertebrates, such as fish [5], birds [23], cattle [24], goats [25], sheep [26], and human [18] with a wide-ranging pattern of tissue expression.

β-defensin genes possess various types of clusters in different species; for example, a single cluster in birds and four clusters in mice, rats, dogs, and cattle, and five in humans and primates. A number of β-defensin genes also vary from species to species, such as in chicken (14), pig (29), dog (38), sheep (43), mouse and human (48), chimp (33), and 57 in bovine [[27], [28], [29], [30]]. They are structured into three key clusters, including 8p23.1, 20p13, and 20q11.1 in humans and a small cluster of chromosome 6p12 [30]. In bovine, a synteny analysis on 57 β-defensin genes with human genome revealed a total of four different clusters, located on four different chromosomes. The majority of the β-defensin encoding genes, i.e., 30 genes are located on chromosome 27, that spans up to 1.9 mb of the chromosome. The second largest cluster of 18 genes spans 320 kb on chromosome 13. Chromosome 23 and 8 contain 5 and 4 genes, which span 51 and 93 kb, respectively [31]. Previous studies suggested that most of the β-defensin genes clustered within the chromosome 27 are expressed mainly in the epithelial tissue and immune cells of the bovine rumen and are associate with the development of the rumen. In addition, the expression of these genes and their phenotypic roles are thought be supported by the “niche adaptation hypothesis” that claims that evolution of the bovine rumen development requires subsequent immune surveillance to cope up against the pathogenic insults. Again, genes that are located on chromosome 13, are preferentially expressed in reproductive organs, that are thought be aided in the rumen development [31]. From early studies on human and Macaque orthologues of protein products β-defensin genes on chromosome 13 suggested that glycosylation of these proteins in sperm could be an important post-translational modification to gain entry through the cervical mucus, protection of the sperm from female immune system, and binding to the oviductal epithelium [31,51]. Expression profile of the β-defensin genes located on chromosome 8 and 23 have not been investigated yet, but the rat orthologues of chromosome 8 gene clusters have been found to be expressed in rat epididymis, while the human orthologues of chromosome 23 gene clusters have been identified in reproductive tract and epithelial cells [31].

β-defensin-like molecules were initially detected from cattle [32]. The eighteen complete and partial bovine beta-defensin sequences were identified through a combination of genome sequence studies and directly isolated from neutrophil [32]. The expression sites of three defensins, appear to be significant variances. The extension of β-defensin HDPs may hold significant potential for fighting against infectious agents and brings opportunities to attach their functions of immune and reproduction of cattle productions.

The comprehensive bioinformatics studies of the bovine genome detected 57 β-defensins in cow [31]. The availability of genomic information has allowed the comparative study and description of β-defensin ranges among numerous species, including humans, rats, dogs, and chickens [17]. Although the role of β-defensins in immune responses against infectious agents and reproduction could be significant, inadequate genomic information is available to explain the whole β-defensin repertoire in cow. There is no previous report on domain or motif based evolutionary and functional analyses of bovine defensin protein. In addition, how do defensins possess that magnitude of functions on the immune system is still not clear. The aim of the present study was, therefore, to investigate the sequence divergence and domain-based evolutionary relations of bovine defensin with that of humans. Our *in silico* study has predicted the presence of different disulfide connectivity, and multiple domains and motifs in β-defensin proteins, which render to the structural and functional variations of these proteins. Comparison of deduced amino-acid sequences of the presented β-bovine defensins with structures of human defensins allows speculation about the functional properties of distinct regions within the mature defensin peptide.

## 2 Experimental procedures

### 2.1 Data retrieval

Defensin proteins and defensin proteins like sequences of *Homo sapiens* were retrieved with the keywords “Human” and “defensin” from the UniProt [https://www.uniprot.org/] and NCBI (https://www.ncbi.nlm.nih.gov/) database. *Bos taurus* defensin has been collected from the UniProt using the keywords “Bovine” and “defensin”. In addition, some defensin proteins of *Bos Taurus* were taken from the NCBI database. All the defensin proteins of humans and cow were then manually filtered and finally, 48 human defensin and 64 bovine defensins have been used for the present study. In addition, tBlastn has been carried out against the GCF_002263795.1 assembly to retrieve the gene sequences using the bovine defensin protein sequences.

### 2.2 Physicochemical properties, domains and signature sequences analysis

Physicochemical properties of the bovine defensins were identified using the ProtParam tool of Expasy (https://web.expasy.org/protparam/). InterPro (Version 5.39–77.0) (https://www.ebi.ac.uk/interpro/) was used for the functionality analysis based on domains and other important sites. The patterns and signatures of the proteins have been detected using ScanProsite [33]. In addition, the O-glycosylation and N-glycosylation sites were predicted using NetOGlyc 3.1 and NetNGlyc 1.0 (http://www.cbs.dtu.dk/services/) [34]. Disulphide connectivity was anlayzed using the DiANNA 1.1 webserver (http://clavius.bc.edu/∼clotelab/DiANNA/). DiANNA 1.1 web server analyzes the presence of intramolecular disulfide bonds by a trained neural network. The signal peptide cleavage site was predicted using the SignalP 5.0 server. (http://www.cbs.dtu.dk/services/SignalP/). It detects the presence of signal peptide and its cleavage site by using a deep convolutional and recurrent neural network architecture.

### 2.3 Chromosomal localization, 3D structure prediction and visualization

Chromosomal localizations were revealed by the tBlastn search against *Bos taurus* (cow) Refseq assembly GCF_002263795.1 (ARS-UCD1.2) and for visualization by Phenograms Plot

(http://visualization.ritchielab.org/phenograms/plot). The tertiary structures were predicted using the Phyre2 (Protein Homology/analogY Recognition Engine V2.0) [35]. Finally, the 3D structures of proteins were visualized using PyMOL.

### 2.4 Multiple sequence alignment and phylogeny analysis

Multiple sequence alignment of the retrieved sequences was performed using the MAFFT alignment (V7.455). We used –auto option for the multiple sequence alignment. In this analysis, “globalpair” was allowed with a maximum number of iterative refinements of 1000. Multiple sequence alignment and conserved sequences were visualized using ESPript 3.0 [36].

Before constructing the phylogenetic tree, the Phyutility program (ver2.2.6; http://blackrim.org/programs/phyutility/) was used to prune the unaligned residues lies within the highly variable regions of the aligned protein and coding sequences. Alignment positions with more than 10% gaps were trimmed. To find out the best-fit substitution model of the proteins analyzed, PartitionFinder (ver 2.1.1) was used [37]. Phylogenetic trees were drawn using the RAxML software (v8.2) [38]. And the PROTGAMMAIJTT model was used. RAxML infers maximum likelihood algorithm from alignments. Finally, the Interactive Tree of Life (iTOL; EMBL, Heidelberg, Germany) has been used for the visualization of the phylogenetic trees.

## 3 Result

### 3.1 Chromosomal localization and physicochemical characteristics of bovine β-defensin

Among the 64 bovine defensin proteins, most of the genes encoding these proteins (29) are located on chromosome 27.25 proteins are translated from genes present on chromosome 13. Again, chromosome 23 and 8 contain genes which code for 7 and 3 proteins, respectively (Supplementary file-1).

Physicochemical properties (Supplementary file-1) revealed that the length of the bovine β-defensin ranges from 38 to 192 amino acid long. Here, the lowest number of amino acids containing defensin proteins are B_DEFB1, 8, and 12, while the highest number of amino acids containing protein is B_DEFB123X1. B_DEFB12 has the lowest molecular weight of 4.1kD, while B_DEFB129 has the highest MW of 21.2kD. Isoelectric pH ranges from 4.8 to 11.47.

### 3.2 Domain architecture and functionality

Different β-defensin proteins in *Bos taurus* contain four different types of domains in their structures. These includes β-defensin (IPR025933), β-defensin type (IPR001855), β-defensin 136/42 (IPR035307) and ß/α-defensin (IPR006080). All these four domains span the sequences that contain six conserved Cys residues which form three pairs of intramolecular disulfide bonds to function against many Gram-positive and Gram-negative bacteria, fungi, and enveloped viruses and possess resistance to microbial growth over epithelial surfaces of different organs.

Among 64 β-defensin proteins in *Bos taurus*, 34 proteins contain only β-defensin domain, only one defensin protein (B_DEFB136) contains β-defensin 136/42 domain only. In cow, 21 proteins contain β-defensin type domain; among these, 17 proteins contain another domain, ß/α-defensin that overlap with the β-defensin type domain. However, eight defensin proteins in cow do not contain the InterPro defined identification number, which include DEFB129, DEFB130lX1, DEFB130lX, DEFB132a, DEFB132b, DEFB109lb, DEFB110X, and DEFB134.

Domain analysis, however, revealed a disorder region (136–170) and a short proline-rich region (155–170) located at the C-terminal region of DEFB129 in mobidb-lite entry (Figure not shown). These regions overlap with each other. DEFB130lX1 and DEFB130lX contain a defensin-like super family entry (SSF57392). DEFB132a and DEFB132b contain a 38 amino acid long CDD entry (cd17462), which represent solute carrier organic anion transporter 4 A subfamily of the Major Facilitator Superfamily of transporters. It is speculated that this subfamily of the domain is ubiquitously expressed and change its’ conformation via a rocker-switch type of movement and is involved in a single substrate binding and translocating it across the membrane [39].

### 3.3 Evolutionary pattern reveals four clusters of bovine β-defensin proteins

The phylogeny reconstruction reveals four clusters of bovine β-defensin proteins (Fig 2). The clusters include cluster I to IV. The bovine β-defensin proteins under these four different clusters have been represented in Table 1.

**Figure-1:**
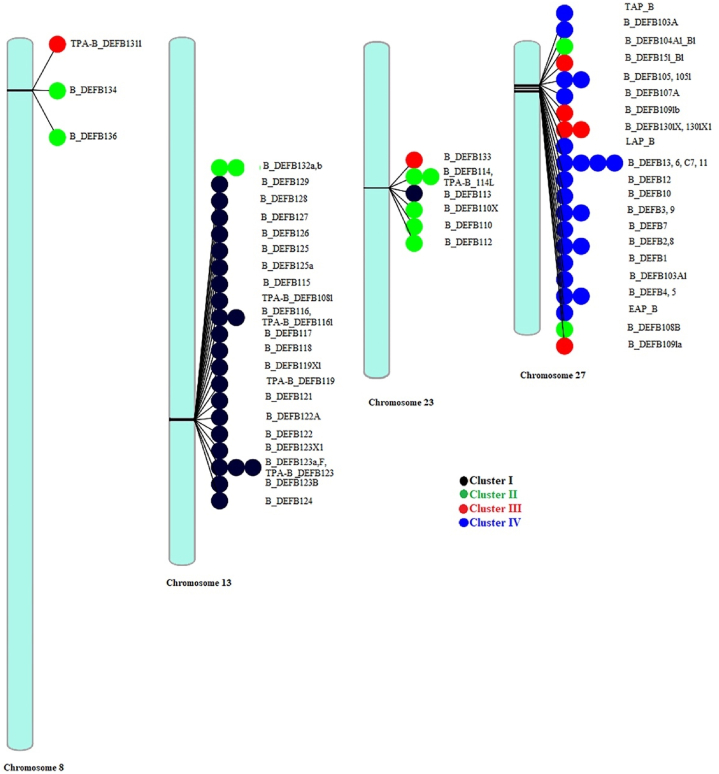
Chromosomal distribution of β-defensin coding genes of *Bos Taurus*. The clusters of β-defensin genes have been shown by different colors. (For interpretation of the references to color in this figure legend, the reader is referred to the Web version of this article.)

**Fig. 2:**
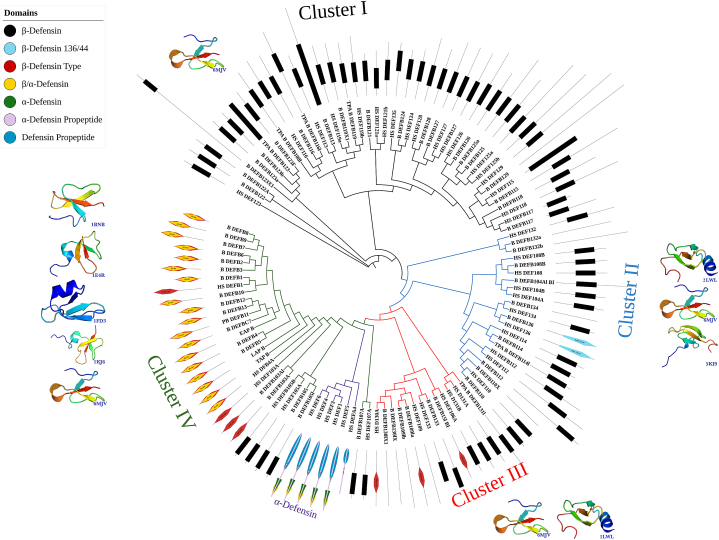
Phylogenetic tree of β-defensin proteins of Human and Bovine. Four different clusters have been shown by different colors. The branches for human α-defensins have been indicated by purple color. Label colors in top left represents domains of proteins. The 3D structures and the PDB IDs of proteins of corresponding clusters have been shown with each cluster. (For interpretation of the references to color in this figure legend, the reader is referred to the Web version of this article.)

**Table-1:** Four different clusters of bovine β-defensin proteins.

Cluster I	Cluster II	Cluster III	Cluster IV
B_DEFB123X1 B_DEFB123a B_DEFB123b TPA_B_DEFB123 B_DEFB123F TPA_B_DEFB108l B_DEFB116 TPA_B_DEFB116l B_DEFB113 TPA_B_DEFB119 B_DEFB119X1 B_DEFB121 B_DEFB124 B_DEFB128 B_DEFB127 B_DEFB126 B_DEFB125a B_DEFB125 B_DEFB115 B_DEFB129 B_DEFB117 B_DEFB118 B_DEFB122 B_DEFB122A	B_DEFB132a B_DEFB132b B_DEFB104Al_BI B_DEFB108B B_DEFB136 B_DEFB134 B_DEFB114 TPA_B_DEFB114l B_DEFB112 B_DEFB110 B_DEFB110X	TPA_B_DEFB131l B_DEFB15l_BI B_DEFB133 B_DEFB109la B_DEFB109lb B_DEFB130lX1 B_DEFB130lX	B_DEFB107A B_DEFB105l B_DEFB105 B_DEFB103A B_DEFB103Al TAP_B LAP_B B_DEFB5 B_DEFB4 EAP_B B_DEFBC7 PB_DEFB11 B_DEFB13 B_DEFB12 B_DEFB1 B_DEFB10 B_DEFB3 B_DEFB2 B_DEFB6 B_DEFB7 B_DEFB9 B_DEFB8

Among the four clusters, Cluster I is the largest cluster that contains 24 proteins, while the Cluster III is the smallest cluster containing 7 proteins. Cluster II and IV contain 11 and 22 defensin proteins, respectively.

Our domain-based phylogeny (Fig-2) clearly indicates variations in domains in different clusters.

Within Cluster-I, we found only the β-defensin domain. We also found isoforms of several defensin proteins of cow in this cluster.

In cluster I, we found two isoforms of DEFB122 of cows grouped together. There are five copies of bovine DEFB123 which form a single group. Among these five copies, the length of DEFB123X1 is unusually long. Again, the β-defensin domain spans the entire length of the TPA DEFB123 and its length is larger than the same domain of other isoforms. Interestingly, the midpoint rooted phylogenetic tree shows that human DEFB123 is placed as an ancestral defensin protein of all the β-defensin proteins of cow and human and, the five copies of bovine DEFB123 do not form a cluster with that of human orthologue. All the other proteins within cluster I form a sub-cluster and split into two other groups. Here, in one group, bovine DEFB108, DEFB116, DEFB113, DEFB119, and their multiple copies are present. The length of bovine DEFB113 is comparatively higher with an unusually long β-defensin domain. In another group of cluster I, it is found that bovine β-defensin protein (and their isoform if present) cluster together with the corresponding orthologues of humans. However, no orthologues of DEFB135 in cow have been found during data retrieval. One unique finding in this group is the absence of the β-defensin domain B_DEFB129, while it is present in HS_DEFB129. This could be happened due to two reasons. Firstly, the domain might not be annotated for this protein, secondly, mutation or SNP might result in loss of the domain feature. In cluster II, we found two sub-cluster. In the small sub-group, B_DEFB132 and its isoform grouped with the human orthologue and no domain has been observed in either of the copies of bovine defensin here. The large group has been split into two; here the bovine defensin proteins with their isoform(s) have been clustered together along with the orthologues from human defensin. Strikingly, in cluster II, all but DEFB136 in cow and its orthologue in humans both contain β-defensin 136/42 domain. In addition, one of the isoforms of B_DEFB110 does not have any conserved domain. Strikingly, Cluster III shows proteins that contain either β-defensin type or β-defensin domain except for DEFB109lb and two isoforms of DEFB130 of bovine. *In silico* analysis could not reveal any domain feature in these later three defensins. Within cluster III, the majority of the proteins contain the β-defensin domain, which indicates that the β-defensin type domain has been evolved from the β-defensin domain. Another interesting finding within this cluster is the variation of domains within defensin 109 homologues, one of the isoforms in cows that possess the newly evolved β-defensin type domain. Cluster IV contains defensin proteins that possess any of the three domains of β-defensin, β-defensin type or ß/α-defensin. Here only DEFB105 and its isoform contain the first domain, and DEFB103 isoforms contain the second domain. The rest of the bovine defensins in this cluster contain both the β-defensin type and ß/α-defensin domains. These domains might have evolved during the natural selection process and involved in different mechanisms of defensin actions in *Bos taurus*.

### 3.4 3D structures support variations within clusters

**Fig. 3:**
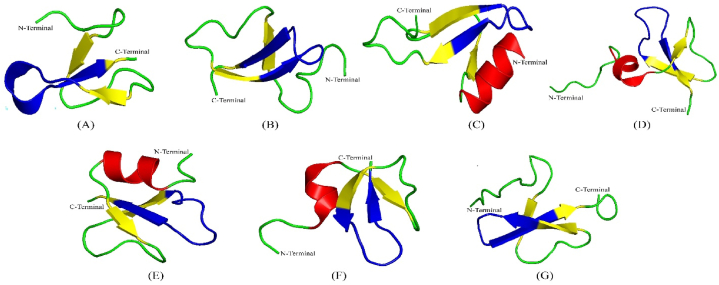
3D structure of bovine β-defensin proteins showing the orientation of the γ-Core. The corresponding PDB IDs are A) 6MJV, B)1BNB, C) 2LWL, D) 1KJ6, E) 1FD3, F) 1E4R, G) 5KI9. The α-helix, β-sheet and chain have been represented by red, yellow and green colour. The γ-Core has been shown by blue colour. (For interpretation of the references to color in this figure legend, the reader is referred to the Web version of this article.)

The cluster variation is further supported by the 3D structures of the proteins (Fig-2, Supplementary file-1). Especially, the presence and length of α-helix has been remarkably changed throughout the evolution (Fig. 2, Fig. 3). We found that proteins of cluster I possess only one type of 3D model, while different proteins in other clusters exhibit significant variations within the same cluster in terms of the 3D models.

Interestingly, the PDB ID: 6MJV that represents mainly the largest cluster I (Fig 2) could not develop a complete α-helical structure rather contain only a very short helical turn; one of whose ends has been fused to chain structure at the N-terminal, and other end has been connected to the β3 sheet at C-terminal (Fig 3A). This structure is somehow different from other 3D models, because all the other proteins either lack the α-helix or, if present, it is seen at the N-terminal of defensin. The 6MJV is the only structure where the incomplete α-helical structure is present in between β2 and β3-sheets. The γ-core has started after the β2 sheet and extended throughout the short α-helix and finished at the β3 sheet (Fig 3A).

Proteins in cluster II possess three types of 3D structures including 6MJV, 2LWL, and 5KI9. The 2LWL contains the standard α-helix at N-terminal and 3 β-sheets (Fig 3C). The γ-core spans some part of β2-sheet, a loop and β3-sheet. Proteins with the PDB ID: 5KI9 contains only three β-sheets, chain, and loop but no α-helix in its’ structure.

Cluster-III defensin proteins contain 6MJV and 2LWL as the 3D representatives. Cluster IV proteins contain four new 3D structures apart from the 6MJV; these are: 1KJ6, 1FD3, 1E4R and 1BNB. All the proteins but 1BNB contain a α-helix, and three β-sheets with a conserved γ-core flank with β2 and β3-sheets. 1BNB, however, lacks the α-helix and contains a long chain at the amino terminal (Fig 3). Some 3D models, however, have been excluded from the study due to either low confidence, low coverage, or both.

### 3.5 Signature patterns and disulfide connectivity

**Figure-4:**
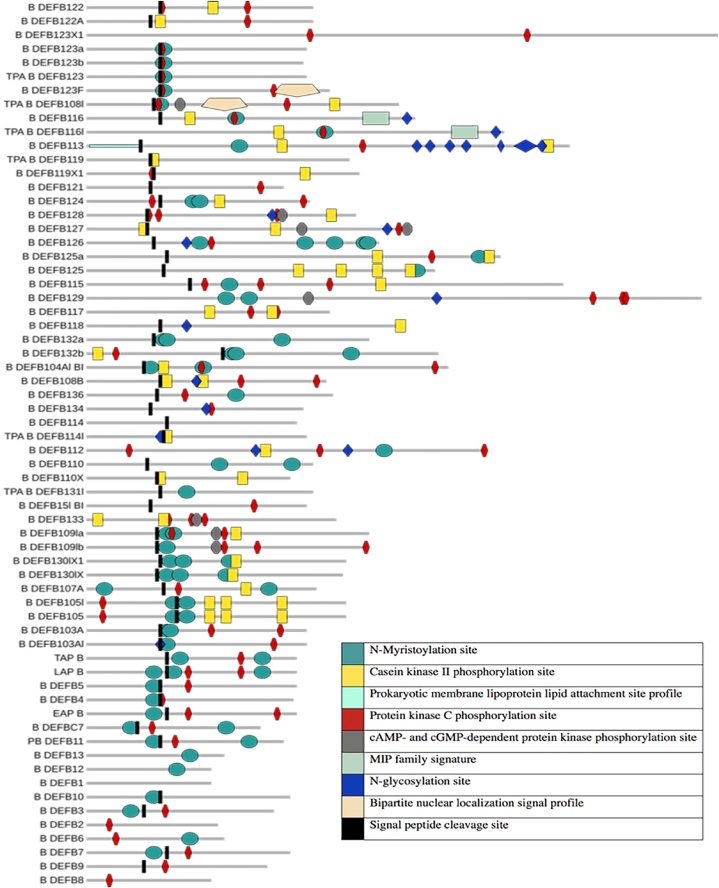
Positions of different patterns and signal peptide cleavage. Name of the patterns have been shown in the right. Protein has been given in grey color with a highest length of 192 amino acids. (For interpretation of the references to color in this figure legend, the reader is referred to the Web version of this article.)

ScanProsite analysis reveals the presence of multiple patterns within different β-defensin proteins of *B. taurus* (Fig 4). The patterns which are found in the defensins are N-glycosylation site (Prosite entry: PS00001), cAMP- and cGMP-dependent protein kinase phosphorylation site (PS00004), Protein kinase C phosphorylation site (PS00005), Casein kinase II phosphorylation site (PS00006), N-Myristoylation site (PS00008), Bipartite nuclear localization signal profile (PS50079), MIP family signature (PS00221) and Prokaryotic membrane lipoprotein lipid attachment site profile (PS51257). These patterns are either overlapping with each other or with different domains. Most of the proteins contain either the N-myristoylation site, casein kinase II phosphorylation site, or protein kinase C phosphorylation site. Prokaryotic membrane lipoprotein lipid attachment site profile is present only in B_DEFB113 and it contains the highest number of N-glycosylation sites. Again, only B_DEFB123F and TPA_B_DEFB108l possess bipartite nuclear localization signal profile within their sequences. In contrast, B_DEFB1 is the only defensin that does not contain any signature sequences. Finally, some but not all the β-defensin proteins of *Bos taurus* contain signal peptide. The signal peptide cleavage site has been shown in Fig 4. In addition, different analyses on the O-glycosylation site revealed that at least 25 bovine defensin proteins contain this site (Supplementary file-1).

**Figure-5:**
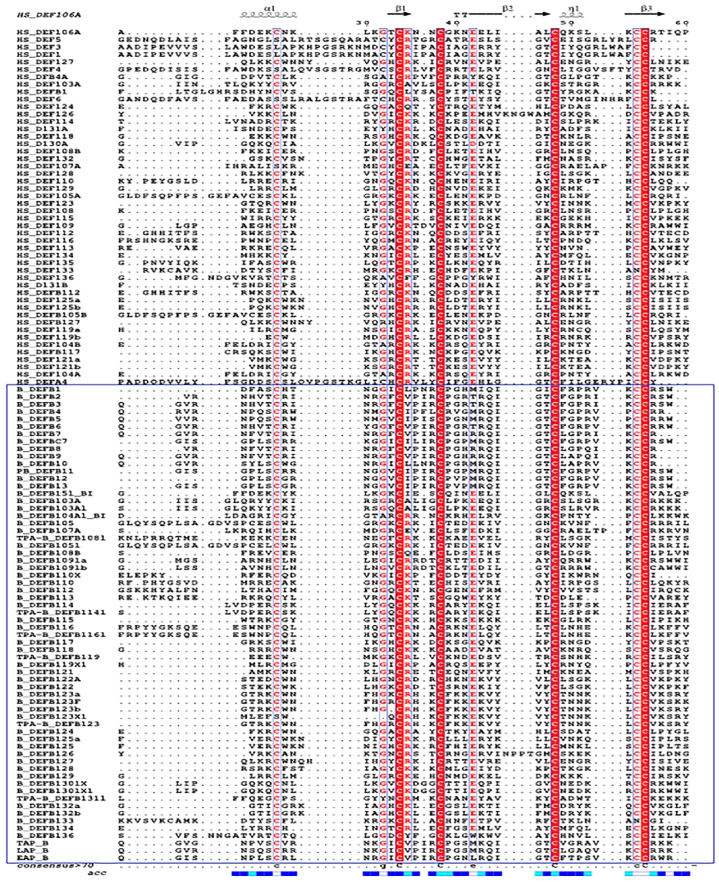
Multiple sequence alignment of defensin proteins of human and bovine. Bovine defensin proteins have been indicated within red color rectangle. The conserved disulfide linkages and other residues have been indicated. Accessibility has been shown below the figure. (For interpretation of the references to color in this figure legend, the reader is referred to the Web version of this article.)

Later, we analyzed the disulphide connectivity of the β-defensin proteins of *Bos taurus* and we found different types of connectivity between the internal Cys residues. 16 defensin proteins including DEFB1, 2, 3, 4, 5, 6, 7, 8, 9, 10, 12,13, PB_DEFB11, DEFBC7, EAP and LAP possess the C1–C5, C2–C4, C3–C6 bond. Our computational analysis revealed four disulphide bonds in six β-defensin proteins of *Bos taurus* (Fig 5). Other disulphide bonds have been presented in Supplementary file-1. Our analysis, however did not get the C1–C6, C2–C4, C3–C5 intramolecular disulphide linkage which is the characteristics of alpha defensin [40].

The 3D structure prediction revealed that 44 bovine defensin proteins showed high confidence (>60%) and medium to high coverage (31–94%) (Supplementary file-1). We found seven different 3D models at this confidence and alignment coverage (Fig 2). Four more 3D structures have been found with high confidence but low alignment coverage.

## 4 Discussion

Our bioinformatics study on 64 members containing the β-defensin family of bovine has aimed to reveal a domain-based evolutionary pattern and functionality prediction.

Chromosomal localization analysis represents that the β-defensin proteins coding genes are densely clustered in four syntenic chromosomal regions, where the majority of the β-defensin protein-coding genes are present on chromosomes 27 and 23. Two small clusters of protein coding genes are present on chromosome 23 and 28 (Fig 1). The presence of gene copy number variation (CNV) endorses that *Bos taurus* might be highly predisposed to a number of diseases [40]. In addition, transcript variants encoding different isoforms have been found which are located on chromosomes 13, 23 and 27 (Fig-1). This CNV has been defined as the result of non-allelic homologous recombination between repeat units. The CNV of defensin is also associated with spatiotemporal expression of defensin genes and functional complexity, and this is particularly achieved via increased level of gene expression, production of novel fusion genes, extending the distance between a gene and regulatory element, or production of new domains with new functionality [55]. However, it should be stressed that this CNV largely depends not only on different bovine species, but also different breeds [56].

Our evolutionary analysis based on human and bovine β-defensin proteins suggests that most of the β-defensins are evolutionary conserved, while some of those are conserved only in bovine. That is why the homologues of some but not all of the bovine defensins are found also in humans. The newly evolved β-defensin might be originated by either gene duplication event, strong positive selection pressure, or even conversion which ultimately leads to a diverse gene family of β-defensin and render neofunctionalization that opt toward different profiles of antimicrobial venture [40,41]. Ultimately, they escalate the host's potential to combat opportunistic infections.

Our study revealed four different clusters of β-defensin proteins. Analysis on chromosomal localization of the protein-coding genes unveiled that chromosome 27 is mainly cluster IV specific, while chromosome 13 is the cluster I specific. This reflects that chromosome 13 contains mainly the ancient β-defensin proteins coding genes, while chromosome 27 contains the most recently evolved proteins coding genes. This finding is contradictory to the previous finding of Meade et al. (2014) [42], where they stated that genes that are clustered on chromosome 27 are the ancient cluster of genes. This is because the earlier studies have been carried out based on the comparison of the rates of synonymous and nonsynonymous nucleotide substitution within genes, while the present study is based on proteins that could be evolved throughout a positive selection for diversification in the amino acid sequences.

Domain-based phylogeny revealed the domain variations among different clusters, especially, cluster I and II showing the same β-defensin domains, cluster IV show β-defensin type, and ß/α-defensin domains and cluster III contains a mixture of domains, while containing only a single domain. This might happen because, ancestral bovine β-defensin proteins contain the β-defensin domain; however, throughout the course of evolution and depending on the natural selective pressure, the newly evolved β-defensin proteins have adapted a new mutation that might produce a new domain while retaining the original defensive role with a diversified profile of antimicrobial activity. Again, this cluster (IV) of proteins containing either β-defensin type only or both β-defensin type and ß/α-defensin domains might have evolved to destine distinct defense role in specific organs and respond toward a variety of inflammatory stimuli.

The presence of multiple 3D models of protein in recently evolved cluster further supports the functionality variations. Again, the absence of the α-helical structure from cluster I defensin proteins might indicate that higher-order functions in this group. In addition, the presence of a very short helical structure in three other different clusters of proteins might be consistent with the fact that the characteristics of α-defensin proteins might have been adopted by some β-defensin proteins of these clusters and promisingly harnessed to improve bovine health.

In cluster I at least 11 proteins contain 100 or more amino acids in their structures. Unlike the long defensin proteins which contains a large extended portion at the N-terminal region [40], the unusually long β-defensin proteins contains a large region after the terminal Cys residue. Therefore, these long β-defensins do not belong to the long defensin proteins. Again, the presence of 8 Cys in some of these β-defensin proteins indicates that these proteins belong to C8. It is undoubtedly proved that both C8 and C6 β-defensin proteins are present in *Bos taurus*. Although these C8 defensins were reported to be present mainly in plants, mollusks, and insects and mainly perform antimicrobial functions [40]. *In silico* disulfide linkage prediction analysis revealed that these proteins contain four disulfide linkages in different patterns. Furthermore, the C6 β-defensin also contains 23 distinct types of disulfide connectivity and there is no C1–C6, C2–C4, C3–C5 bond that is specific for α-defensin. 27 disulfide linkages in defensin proteins have been reported previously [40]. Earlier studies suggested the presence of different patterns of intramolecular disulfide bridge connectivity in human defensin derivatives and those confer the peptide stability against proteolytic degradation [42]. In the contrary, different disulfide linkages might also render nonfunctional proteins or even loss of functionality, this requires to be proved by laboratory experiments.

In our study, we found that the γ-core is located centrally within the defensin peptide; however, according to the true definition, the antiparallel β-sheets should form a γ type structure, and this type of structure is either partially present or completely absent in the proteins that shows following 3D model: 1BNB, 1KJ6, 1FD3, and 1E4R. In most of the cases we found β -γ- β structure, while only in 6MJV it is γ-β. Although γ-core is said to be highly conserved in most of the animals [57], in bovine β-defensin, almost 50% proteins exhibit XXC motif instead of GXC motif (Fig 4). Therefore, the shapes and sequences partly support the findings of Yount and Yeaman (2006) on cysteine rich antimicrobial peptide.

Our bioinformatics analysis also revealed specialized patterns within different β-defensin proteins of cow. N-myristoylation site (NMS) is present at least one-third of the defensin proteins in cow and is widely distributed in all four clusters. Some β-defensins even contain more than one NMS but in most cases, these sites might become inactivated as these falls over the signal peptide cleavage site (Fig 4). The myristoylation process is a co-translational lipid modification, catalyzed by the eukaryotic N-myristoyl transferase enzyme. Protein possessing the active NMS is one of the obligatory steps of many important innate immune signaling pathways [43,44]. In an earlier study, it has been shown that in neutrophils following the expression of myristoylated alanine-rich C kinase substrate (MARCKS) owing to the effect of microbial infection and subsequent presence of LPS (lipopolysaccharides), MARCKS undergoes myristoylation. This is important for the accumulation of protein kinase C (PKC) that ultimately mediate the inflammatory signal [43,44]. Likewise, it can be inferred that the presence of both NMC and PKC phosphorylation sites might follow the same mechanism i.e., these proteins might undergo myristoylation at the NMS while sensing the LPS, which might cause the colonization of PKC. Recruitment of PKC causes the phosphorylation at the PKC phosphorylation site of the defensin proteins which finally trigger the downstream immune signaling cascades. In another study on human defensin 1-3 analogs, myristoylation was carried out by increasing the hydrophobicity and these peptides showed enhanced antimicrobial activity against *Staphylococcus aureus* and *Escherichia coli* infection. They suggested that hydrophobicity along with the presence of a different numbers of cationic residues are responsible for myristoylation and a varying extent of hemolytic activity [45]. Again, an *in vitro* study on a myristoylated antibacterial peptide PMAP-36PW (Myr-36PW) showed a significant therapeutic effect against biofilms by increasing the membrane permeability. A further experiment on mouse pneumonia and peritonitis disclosed that the Myr-36PW exhibited promising reducing effect against *Staphylococcus aureus* ATCC 25923 and *Pseudomonas aeruginosa* GIM 1.551 in the infected organ and reduced the abscess and promoted wound healing [46]. Therefore, the NMS of defensin proteins would be a good target for enhancing the defense mechanism especially in drug-resistant bacteria. Subsequently, the DEFB in bovine which possess the NMS sites could be highly active against the drug-resistant bacteria.

The casein kinase II phosphorylation site (CK2PS) pattern is mainly found in those bovine β-defensins, which contain the β-defensin domain in their structure, with the exception of B_DEFB109la, that contain β-defensins type domain. In RNA viral infection, phosphorylation of the RIG-I an RNA helicase containing caspase activation and recruitment domains by CK2 results in ablation of IFN-α/β mediated downstream antiviral response mechanism [47]. This finding is further corroborated by another study, which suggests that CK2 acts as a switch that negatively control the TBK1 and IRF3 activation in IFN-inducing TLR, RIG-I–like receptors, and cGAS/STING signaling pathways [48]. The presence of CK2PS on defensin might, therefore consist with the fact that this site is prone to CK2 mediated phosphorylation which might lessen the effect of antiviral action.

The Prokaryotic membrane lipoprotein lipid attachment site profile which is present in the N-terminal of the B_DEFB113 is a precursor signal peptide. A signal peptidase recognizes and cut the upstream of the Cys residue at position 16 and probably renders an attachment site for the glyceride fatty acid lipids like N-palmitoyl cysteine or S-diacylglycerol cysteine which might anchor this protein to the microbial membrane [49]. This attachment site is present in a number of bacterial proteins. The presence of bipartite nuclear signal profile indicates that B_DEFB113 is uptaken by nucleus [50] and it possesses its defensive role within the nucleus.

MIP family signature has a sequence of NSNPSLSVT, which is an important feature of transmembrane channel proteins. Though *B_DEFB116 and* TPA-B-DEFB116l contain this MIP signature and both of them are clustered together with HS_DEFB116, the later one lacks this signature.

The presence of N-glycosylation site (NGS) in defensin and subsequent binding of carbohydrates is not uncommon as glycosylated defensins and have been found mainly in the reproductive tract in humans [51]. At least eight bovine defensin proteins in cluster I and four proteins in cluster II contain this NGS in their structure. However, only four defensin proteins in cluster I contain NGS in their C-terminal site and B_DEFB113 contains multiple glycosylation sites. Though the computational analysis revealed the presence of the tri-peptide NGS in the bovine defensins, the actual glycosylation depends on the folding structure. In the case of defensin play its protective role in sperm during traveling through the female reproductive tract, the glycosylation site along with the four core Cys residues are important in a folding structure which is mainly present in B_DEFB126. In addition, we found O-glycosylation sites (OGS) in many bovine defensin. A study on bovine DEFB126 revealed the presence of a single NGS and an OGS in its structure and this DEFB126 forms a dissociation resistant dimer to coat caudal sperm and might regulate sperm motility through cervical mucus and give protection to the sperm from attack by the female immune system while traveling toward the oviduct [52]. Therefore, it can be assumed that other bovine defensins which contain both the NGS, and OGS might also function in the reproductive organ.

Expression and activation of different β-defensins vary with the type of infectious agents, tissues, and developmental stages of the target organ. For example, Roosen et al. reported the presence of multiple β-defensins in cluster IV during mastitis, and different candidate defensin genes are inducibly expressed in mammary glands [53]. In addition, he also found some β-defensins in both healthy and infected tissues of mammary glands which indicate both the constitutive and inducible expression of these genes. The constitutive expression of these defensin bolsters the priming procedure of the defensin proteins against microbial attack. We tried to align their outcomes with our present study and found that these defensins including LAP, TAP, DEFB3,7,8,9,10,11 contain either N-myristoylation or protein kinase-C phosphorylation site or both and span overlapping β-defensin type and ß/α-defensin domains.

Bovine β-defensins from different clusters are expressed embryo, oral cavity, saliva, milk, respiratory tract, esophagus, stomach, duodenum, small intestine, large intestine, colon, reproductive tract of both male and female in either diseased or healthy conditions and confer a range of dynamic functions [54]. This indicates the role of β-defensins in both healthy and pathogenic microbiome; the mechanism of action is still unknown and needs in-depth research.

## 5 Conclusion

Our domain-based phylogeny reveals four clusters of bovine β-defensin with diversified functionality with different mechanisms. Although our present study shed light on the presence of domains, motifs and corresponding functions, the computational study could not draw the actual picture of the functionality of bovine defensins and requires the wet lab validation. Again, differential transcriptome analysis to understand the difference between the expression level of the defensin genes and regulatory molecules in different locations e.g., reproductive organs, and blood could give more profound information of organ specific mechanism of different β-defensin proteins in cow. Understanding the actual mechanism of action of β-defensin in target organ will be a step forward toward the development of alternative of antibiotics or vaccine to prime the body with a controlled supply of defensins that will combat against various infectious diseases.

## Author contribution statement

Saiful Islam: Conceived and designed the experiments; Performed the experiments; Analyzed and interpreted the data; Wrote the paper.

Mst Rubaiat Nazneen Akhand: Conceived and designed the experiments; Performed the experiments; Analyzed and interpreted the data; Contributed reagents, materials, analysis tools or data; Wrote the paper.

Mahmudul Hasan: Contributed reagents, materials, analysis tools or data; Wrote the paper.

## Funding statement

This research did not receive any specific grant from funding agencies in the public, commercial, or not-for-profit sectors.

## Data availability statement

Data associated with this study has been deposited at Uniprot and NCBI under the accession number P0DP73; P59861; A0A096LNP1; P59665; P81534; Q8WTQ1; AAI00850.1; Q8NG35; Q8N104; Q8IZN7; A8MXU0; Q8NET1; Q30KR1; Q30KQ9; Q30KQ8; Q30KQ7; Q30KQ6; Q30KQ5; Q30KQ4; Q96PH6; Q8N690; Q8N690-2; NP_001165303.1; Q5J5C9; Q8N688; Q8NES8; NP_697020.2; Q8N687; Q9BYW3; Q9H1M4; Q7Z7B8; Q9H1M3; Q7Z7B7; Q30KQ1; Q4QY38; Q30KP9; Q30KP8; P59666; P12838; Q01523; Q01524; A0JDY5; P60022; B2RU30; A0A494C1K0; Q30KQ3; Q14DW6; O15263; XP_19808806.1; XP_27385898.1; XP_27385834.1; XP_19808902.1; XP_24842095.1; XP_2698669.1; XP_27385632.1; XP_27385609.1; XP_27385545.1; XP_24839746.1; XP_24839712.1; XP_24839622.1; XP_24857141.1; XP_10809939.1; XP_24856609.1; XP_24856654.1; XP_5215144.1; XP_5215143.2; XP_24842220.1; XP_24842219.1; XP_5215029.4; XP_24839668.1; XP_24852155.1; XP_24852156.1; P46159; P46160; P46161; P46162; P46163; P46164; P46165; O18815; P46166; P46167; P46168; P46169; P46170; P46171; DAA23405.1; A0A3Q1N0H9; A0A3Q1MB27; G3N236; DAA16482.1; DAA23406.1; AEP81460.1; AEP81459.1; DAA23097.1; A0A3Q1MVX4; A7LM98; Q0VBX1; ABS86889.1; A0JNM7; F6RSW1; DAA23339.1; A7LMA0; AEP81453.1; AEP81452.1; AEP81454.1; AEP81455.1; DAA27089.1; AEP81451.1; P25068; Q28880; O02775.

## Declaration of interest’s statement

The authors declare no conflict of interest.
